# The Development of Dyslipidemia in Chronic Kidney Disease and Associated Cardiovascular Damage, and the Protective Effects of Curcuminoids

**DOI:** 10.3390/foods12050921

**Published:** 2023-02-22

**Authors:** Zeltzin Alejandra Ceja-Galicia, Ana Karina Aranda-Rivera, Isabel Amador-Martínez, Omar Emiliano Aparicio-Trejo, Edilia Tapia, Joyce Trujillo, Victoria Ramírez, José Pedraza-Chaverri

**Affiliations:** 1 Department of Cardio-Renal Physiopathology, National Institute of Cardiology Ignacio Chávez, Mexico City 14080, Mexico; 2 Department of Biology, Faculty of Chemistry, National Autonomous University of Mexico, Mexico City 04510, Mexico; 3 National Council on Science and Technology (CONACyT)-Instituto Potosino de Investigación Científica y Tecnológica-División de Materiales Avanzados (CONACyT-IPICYT-DMA), San Luis Potosí 78216, Mexico; 4 Department of Experimental Surgery, National Institute of Medical Science and Nutrition Salvador Zubirán (INCMNSZ), Mexico City 14080, Mexico

**Keywords:** curcumin, curcuminoids, chronic renal disease, cardiovascular disease (CVD), dyslipidemia, CKD

## Abstract

Chronic kidney disease (CKD) is a health problem that is constantly growing. This disease presents a diverse symptomatology that implies complex therapeutic management. One of its characteristic symptoms is dyslipidemia, which becomes a risk factor for developing cardiovascular diseases and increases the mortality of CKD patients. Various drugs, particularly those used for dyslipidemia, consumed in the course of CKD lead to side effects that delay the patient’s recovery. Therefore, it is necessary to implement new therapies with natural compounds, such as curcuminoids (derived from the *Curcuma longa* plant), which can cushion the damage caused by the excessive use of medications. This manuscript aims to review the current evidence on the use of curcuminoids on dyslipidemia in CKD and CKD-induced cardiovascular disease (CVD). We first described oxidative stress, inflammation, fibrosis, and metabolic reprogramming as factors that induce dyslipidemia in CKD and their association with CVD development. We proposed the potential use of curcuminoids in CKD and their utilization in clinics to treat CKD-dyslipidemia.

## 1. Introduction

Chronic kidney disease (CKD) is a global public health problem, with an incidence of >11.1% [1], corresponding to 843.6 million cases worldwide [2]. CKD significantly increases cardiovascular morbidity and mortality rates since CKD increases cardiovascular events by more than 50% [3,4,5,6]. Several risk factors are shared between CKD and cardiovascular disease (CVD), including diabetes, hypertension, lipid abnormalities, obesity, and smoking. CKD-induced dyslipidemia has been highlighted as a critical factor in CVD development [7,8]. CKD patient management involves using different drugs to reduce cardiovascular risk and prevent renal venous hypertension and congestion. These drugs include antihyperlipidemic combinations, renin-angiotensin-aldosterone system (RAAS) inhibitors, angiotensin receptor blockers, diuretics, vasodilators, inotropes, and β-blockers [8,9,10]. However, it has been reported that these drugs might cause side effects, doing more challenging to treat CKD patients [8]. Therefore, new treatment strategies are required to avoid or reduce dyslipidemia in CKD and the associated CVD without these side effects.

Curcuminoids are compounds derived from turmeric (*Curcuma longa*) root, used in traditional medicine and as a pigment, additive, and spice for several years [11]. In CKD, curcuminoids have received significant interest due to their several health-beneficial properties, such as antioxidative, anti-inflammatory, antifibrotic, and others [12]. In addition, it has been hypothesized that curcuminoids can reduce dyslipidemia in CKD; however, the beneficial effects of curcuminoids on dyslipidemia in CKD and associated CVD are poorly explored. Therefore, this review aims to describe some mechanisms that lead to dyslipidemia in CKD and how these mechanisms promote CVD development. We also discuss the use of curcuminoids to attenuate CKD-induced dyslipidemia and the associated CVD. 

## 2. Pathophysiological Features of Dyslipidemia in CKD

CKD is a chronic disorder characterized by kidney structure and function abnormalities for 3 months or more [13]. CKD is classified into five stages based on the estimated glomerular filtration rate (eGFR), serum creatinine, and albuminuria levels [14,15]. The advanced stages of CKD are characterized by a decreased eGFR of less than 60 mL/min per 1.73 m^2^, which leads to progressive glomerular, tubular, and interstitial damage [13]. Several etiologic factors predispose to CKD development, including diabetes, hypertension, vascular disease, and glomerulonephritis [16]. Mechanistically, the pathophysiology of CKD is characterized by overstimulation in the RAAS, oxidative stress, inflammation, fibrosis, and dyslipidemia [14,17]. Dyslipidemia is an unfavorable lipid profile that occurs in approximately one-third of patients [18], complicating their treatment [16,19]. Dyslipidemia results from the imbalance of lipids such as cholesterol, triglycerides, and lipoproteins. Lipoproteins are macromolecules that transport lipids into the bloodstream to deliver them to the organs [20]. These macromolecules are synthesized in the liver and are denominated according to their density as high-density lipoprotein (HDL), low-density lipoprotein (LDL), and very-low-density (VLDL). VLDL and LDL mainly transport triglycerides to the tissues, while HDL transports cholesterol back to the liver [21]. 

Patients with CKD develop dyslipidemia since the early stages of renal dysfunction [19], which may increase the CKD progression rate [22]. Dyslipidemia in CKD is characterized by elevated triglycerides, cholesterol, VLDL, LDL, and low concentrations of HDL [23]. Furthermore, the size of LDL tends to be smaller and denser, related to atherogenic risk. The levels of apoproteins (proteins associated with lipoproteins) are also altered, characterized by the decrease of HDL apolipoprotein A1 (apoAI) and the accumulation of cholesterol. More specifically, CKD patients with dyslipidemia have elevated total cholesterol (above 240 mg/dL) and LDL-cholesterol levels (above 130 mg/dL), reduced HDL-cholesterol, and increased LDL-cholesterol/HDL-cholesterol ratio [24]. These alterations have been linked to the decrease in renal clearance and the altered enzyme activity of lipoprotein lipase, which induce alterations in triglyceride elimination [25]. In addition, the apolipoprotein B (apoB)/apoAI ratio is higher, causing the increase of LDL in plasma [26]. Therefore, dyslipidemia is a progressive disease with the potential need for additional lipid-lowering modifications in CKD patients [27]. Moreover, several studies have shown that dyslipidemia during CKD might lead to CVD development [7].

## 3. Dyslipidemia in CKD-Induced Cardiovascular Damage 

CVD is a growing condition that produces high rates of mortality and disability in the world [28,29]. CKD is an independent risk factor for CVD development, and CVD degree closely correlates with CKD severity [5,30]. For example, patients with an eGFR less than 60 mL/min/1.73 m^2^ have a three-fold increased risk of heart failure (HF) [31], while patients with end-stage renal disease or on dialysis have a 10–30-fold increased risk of cardiovascular events for all-cause [10,17]. In addition, the lipid accumulation in plasma causes atheroma, increasing the CVD risk [19].

It is well-recognized that dyslipidemia is a risk factor for CVD [32]. In the clinical context, two profiles are considered to determine dyslipidemia. In the first, total cholesterol, LDL, triglycerides, and apoB levels are above the 90th percentile of the general population. In the second, HDL and apoA1 levels are below the 10th percentile of the general population [33,34]. Low HDL levels have been associated with a significant risk factor for chronic and ischemic heart disease [35]. An updated statistical report from the American Heart Association in 2020 showed that 38% of the adult population had elevated triglyceride levels (>200 mg/dL), and 29% had elevated LDL levels (>130 mg/dL) [36]. The alterations in these lipids are a risk factor for the development of dyslipidemia. Dyslipidemia also produces alterations such as atherosclerotic CVD [37], which is the leading cause of death and disability in the elderly [38]. The patient’s condition worsens if other factors, such as smoking, body weight, hypertension, and diabetes, are involved [39]. In addition, atherosclerosis induces endothelial damage, leading to inflammation and the production of a fibrotic plaque that inhibits lipid metabolism [37]. 

The development of dyslipidemia is a common factor between CKD and CVD, which complicates the illness and its progression [26]. In clinical trials, it has been shown that the reduction in LDL-cholesterol levels in CKD patients is directly proportional to the decrease in CVD risk [40,41]. Dyslipidemia increases the risk of developing atheroma and arteriosclerotic plaques. These injuries increase vessel thickness and decrease resistance, promoting blood pressure changes. Moreover, the vessels can develop aneurysms, increasing the risk of internal bleeding and death [42]. Therefore, CVD because of dyslipidemia continues to be a factor that contributes to higher mortality and morbidity in CKD patients. CKD causes a systemic and permanent proinflammatory state that contributes to vascular and myocardial remodeling processes, vascular senescence, and myocardial fibrosis [43]. CKD patients manifest cardiovascular outcomes as coronary artery disease, HF, arrhythmias, and sudden cardiac death [43]. In addition, metabolic changes have also been proposed [44,45,46]. Although several guidelines exist to guide healthcare providers in treating dyslipidemia, there are no specific recommendations for the CKD population [19]. Furthermore, some preventive therapies to reduce lipid levels in patients with CKD, such as statins and other drugs, are not always optimal for treating CKD patients [18]. Thus, strategies to improve some of these symptoms could be crucial in treating CKD-induced dyslipidemia and the associated CVD. 

## 4. Mechanisms Involved in CKD-Induced Dyslipidemia and Associated Cardiovascular Damage

### 4.1. Oxidative Stress

Oxidative stress is recognized as an imbalance between the production of reactive oxygen species (ROS) and their elimination. In the kidney, the primary ROS sources are mitochondria, nicotinamide adenine dinucleotide phosphate hydrogen (NADPH) oxidases (NOX), peroxisomes, and endoplasmic reticulum; however, the main contributors in CKD are NOX and mitochondria, mainly in the tubular segments of the nephron [47,48]. Although mitochondria canonically generate 1–3% of electron leakage, inducing low ROS production, mitochondria dysfunction produces ROS in large amounts during CKD [49]. Furthermore, ROS produced by NOX is upregulated in CKD, which increases oxidative stress [50].

**Figure 1:**
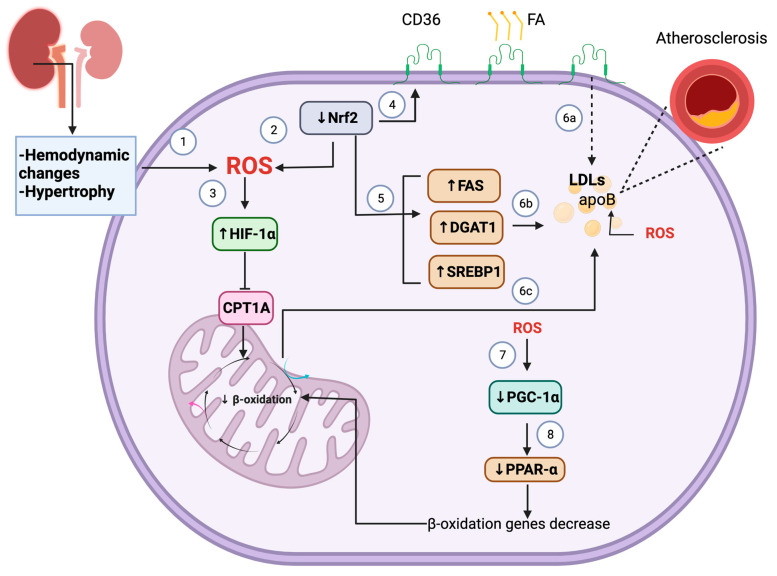
Oxidative stress causes dyslipidemia. (1) Hemodynamic changes and hypertrophy increase reactive oxygen species (ROS) levels. (2) Moreover, the downregulation of nuclear factor erythroid 2-related factor 2 (Nrf2) leads to ROS overproduction, which activates hypoxia-inducible factor (HIF)-1α. (3) The activation of HIF-1α causes the inhibition of carnitine palmitoyl transferase 1 A (CPT1A), the rate-limiting enzyme of β-oxidation in mitochondria, inducing the downregulation of β-oxidation. (4) The downregulation of Nrf2 also causes the increase of cluster of differentiation 36 (CD36), which augments fatty acid (FA) uptake. (5) Furthermore, the decrease of Nrf2 boosts the levels of enzymes involved in FA synthesis, such as FA synthase (FAS), diacylglycerol O-acyltransferase 1 (DGAT1), and sterol regulatory-element binding protein 1 (SREBP1). (6a) The increase in CD36 levels, (6b) the upregulation of FA synthesis enzymes, and (6c) the downregulation of β-oxidation lead to the accumulation of low-density lipoproteins (LDLs). LDLs can be oxidized via ROS into apolipoprotein B (apoB), contributing to atherosclerosis. (7) ROS also downregulates peroxisome proliferator-activated receptor γ co-activator 1α (PGC-1α), which influences (8) the decrease of peroxisome proliferator-activated receptor-alpha (PPAR-α), leading to β-oxidation genes decrease.

During CKD, hemodynamic changes and hypertrophy induce ROS overproduction, which might activate hypoxia-inducible factor (HIF)-1α, triggering lipid accumulation (Figure 1). This mechanism implies the repression of carnitine palmitoyl transferase 1 A (CPT1A), the rate-limiting enzyme of β-oxidation in mitochondria [51]. Additionally, high ROS levels promoting oxidative stress led to peroxisome proliferator-activated receptor γ co-activator 1α (PGC-1α) deactivation, downregulating β-oxidation, and contributing to fatty acid (FA) accumulation [52]. Indeed, dyslipidemia might be caused by oxidative stress due to high ROS levels leading to PGC-1α deactivation, downregulating β-oxidation, and contributing to FA accumulation [52]. This mechanism could be explained since PGC-1α interacts with peroxisome proliferator-activated receptor-alpha (PPAR)-α to regulate FA metabolism through genes involved in β-oxidation [52]. Thus, the dysregulation of PGC-1α leads to the downregulation of β-oxidation genes, inducing a decrease in FA oxidation into mitochondria (Figure 1). Additionally, ROS overproduction might lead to the oxidation of lipids in the membranes, which further increases cell damage [53].

Another protein affected by ROS is nuclear factor erythroid 2-related factor 2 (Nrf2), which is commonly downregulated in CKD [54]. In contrast, the levels of Kelch-like-ECH associated protein-1 (Keap-1), a negative regulator of Nrf2, are upregulated, possibly contributing to low levels of Nrf2 [55]. The decrease in Nrf2 has been related to FA metabolism alterations. In line with this, in type 2 diabetes, the low levels of CPT1A and acetyl-CoA carboxylase (ACC) were rescued by sulforaphane (SFN), a potent Nrf2 activator, suggesting that Nrf2 reduction decreases the levels of these proteins [55]. Supporting this, our group has recently reported that SFN alleviated FA metabolism dysfunction in the unilateral ureteral obstruction (UUO) model by downregulating cluster of differentiation 36 (CD36) levels and the FA synthesis proteins, such as FA synthase (FAS), sterol regulatory-element binding protein 1 (SREBP1) and diacylglycerol O-acyltransferase 1 (DGAT1), as well as triglyceride levels in the renal tissue [56]. These data suggest that the restoration of Nrf2 results in improving lipid metabolism impairment in the renal damage caused by obstruction. Interestingly, Nrf2 overactivation might have deleterious consequences in dyslipidemia [57]. This hypothesis is sustained due to Nrf2 regulating the transcription of CD36 by positioning in its promoter region. Following the latter, in a model of atherosclerosis, the upregulation of Nrf2 leads to the transcription of CD36, which causes free-cholesterol accumulation due to the presence of high levels of FA [58]. However, additional studies are required to determine the effect of Nrf2 overactivation in other kidney disease models.

Oxidative stress promotes atherosclerosis by modifying the lipoproteins and proteins involved in FA metabolism. For instance, intermediate LDL and LDL are accumulated in uremia, which causes the oxidation, carbamylation, or glycation of apoB contained in these lipoproteins [59]. The oxidation of LDL-cholesterol produces oxidized (Ox)-LDL-cholesterol. 4-hydroxy-2-nonenal (4-HNE) is the most abundant aldehyde in Ox-LDL-cholesterol and malondialdehyde (MDA); MDA has been found in the plasma of CVD patients [59]. 

The accumulation of Ox-LDL-cholesterol can also damage the mitochondria, increasing the leakage and the subsequent production of ROS and later oxidative stress [60]. Additionally, macrophages induce Ox-LDL-cholesterol uptake, forming macrophage foam cells in the walls of the vessels, which also cause even more oxidative stress. In this way, oxidative stress promotes atherosclerotic plaque development [60].

In summary, hemodynamic changes and hypertrophy induce ROS, causing the inactivation of Nrf2. The decrease of Nrf2 decreases β-oxidation through the activation of HIF-1α. Moreover, Nrf2 low levels increase CD36 expression and the levels of FA biosynthesis enzymes. ROS also deactivates to PGC-1α, promoting the downregulation of β-oxidation genes. These alterations lead to LDL accumulation and oxidation. Ox-LDLs are the leading factors for atherosclerotic lesions development (Figure 1). 

### 4.2. Inflammation and Fibrosis

Inflammation is present during CKD, supporting kidney damage through the release of cytokines, chemokines, and other molecules that lead to the recruitment of macrophages, neutrophils, and lymphocytes to the damage site [61]. These inflammatory cells secrete additional molecules, inducing a vicious cycle that contributes even more to damage. Fibrosis is a part of the repair process that develops in response to injury. However, the dysregulation of fibrosis causes an overproduction of extracellular matrix proteins, mainly collagen [22,62]. In kidney diseases, both inflammation and fibrosis go hand in hand. For example, secretion of tumor necrosis factor (TNF)-α, an activator of nuclear factor-kappa B (NF-κB), results in the production of transforming growth factor (TGF)-ß by fibroblasts [63].

In the same way, in renal interstitial fibrosis, the infiltration of inflammatory cells, mainly lymphocytes and macrophages, promotes fibrosis through the M2 CD206^+^ phenotype, leading to various degrees of renal failure [64]. In addition, macrophages can undergo the macrophage-to-myofibroblast transition process, contributing to the fibrotic process [65]. Thus, inflammation and fibrosis are CKD’s leading causes of kidney damage. 

**Figure 2:**
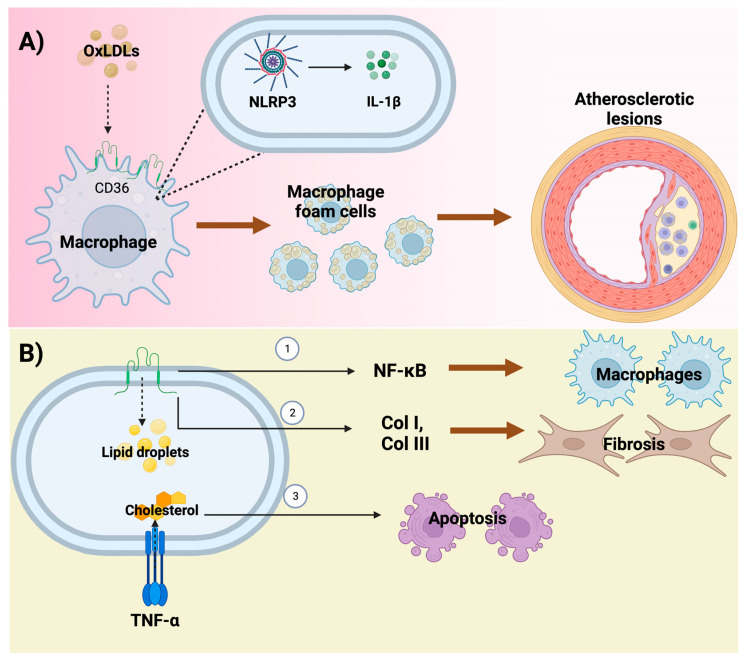
Effect of inflammation and fibrosis in chronic kidney disease (CKD)-induced dyslipidemia. (**A**) The presence of the cluster of differentiation 36 (CD36) in macrophages promotes the uptake of oxidized low-density lipoproteins (Ox-LDLs), which activates nucleotide-binding oligomerization domain-like receptor-containing pyrin domain 3 (NLRP3), inducing the secretion of IL-1β and the later the formation and accumulation of foam cell, leading to atherosclerotic lesions. (**B**) CD36 also (1) activates nuclear factor-kappa B (NF-κB), which leads to the recruitment of macrophages. Additionally, CD36 (2) promotes fibrosis by promoting the overexpression of collagen I (Col I) and collagen III (Col III). TNF-α induces cholesterol accumulation, which triggers (3) apoptosis of renal cells.

Inflammation and fibrosis are closely related to metabolic disorders such as dyslipidemia [66]. This relationship is observed through CD36, an integral membrane protein that not only facilities FA uptake but is also related to inflammation and fibrosis [67]. Furthermore, this receptor is expressed in macrophages, inducing the capture of ligands such as apoAI, lipopolysaccharide, FA, and Ox-LDL [68]. A study reported that the overexpression of CD36 on macrophages contributes to foam cell formation and subsequent accumulation, leading to atherosclerotic lesions (Figure 2) [69]. These mechanisms are triggered primarily by CD36 increasing Ox-LDL consumption, which then induces interleukin (IL)-1ß secretion mediated by activation of the nucleotide-binding oligomerization domain-like receptor containing pyrin domain 3 (NLRP3) [69]. Furthermore, in hypercholesterolemia-induced CKD, the deletion of CD36 decreases NF-κB, preventing interstitial macrophage infiltration [70]. Additionally, CD36^-/-^ mice showed less fibrosis compared to CD36 wild type, suggesting that the decreased lipid accumulation could prevent inflammation and fibrosis in this model [70]. Therefore, CD36 increase and overactivation promote the maintenance of inflammation and fibrosis in CKD models. Interestingly, the upregulation of CD36 in rodent models has been related to CVD caused by type II diabetes, obesity, and insulin resistance [71,72].

The association between inflammation and dyslipidemia has also been linked through TNF-α in a diabetic nephropathy urine model where the injection of TNF-α caused the accumulation of cholesterol and favored apoptosis [73]. This study indicates that inflammatory markers promote the dysregulation of lipid metabolism. In addition, the cytokine tumor necrosis factor-like weak inducer of apoptosis, a member of the TNF-α family, induces PGC-1α downregulation via NF-κB [74], suggesting that β-oxidation might be altered due to the upregulation of inflammatory pathways.

### 4.3. Metabolic Reprogramming

The kidneys are highly energy-demanding organs [75,76], and the mitochondria principally sustain adenosine triphosphate (ATP) production in these organs to carry out the reabsorption process [77,78,79]. The principal substrates used by kidneys are FA, metabolized via β-oxidation [80,81,82,83]. In contrast, glycolytic pathway contribution is strongly limited under normal physiological conditions [84,85,86]. Mitochondrial dysfunction is a common pathology observed in several types of CKD [75,76,87]. In CKD, the activation of lipogenesis pathways decreases β-oxidation and mitochondrial biogenesis through PGC-1α and PPAR-α reduction [88]. In this context, mitochondria fail to respond to the CKD-induced ATP demand increase [87,89,90], which induces a metabolic reprogramming characterized by the shift from mitochondrial-based to anaerobic metabolism [91,92]. Additionally, the increase in triglyceride synthesis and FA uptake proteins has been observed since the early stages of CKD [76,93], favoring lipid deposition in nephrons [92,93,94]. Likewise, FA release from phospholipids is also stimulated [76]. Therefore, metabolic reprogramming has been suggested as a critical factor that allows dyslipidemia in CKD [76,95,96]. CKD patients and experimental models have shown that elevated protein levels of CD36 indicate an increase in lipid uptake [97]. CD36 also increases PPAR-γ abundance and produces positive feedback increasing CD36 and FA binding protein (FABP), favoring the lipid droplets formation and their later accumulation in the kidneys [97]. According to the latter, dyslipidemia also is developed in the nephrectomy model due to metabolic reprogramming, which increases FA synthesis and decreases mitochondrial β-oxidation in the kidney [98,99].

In experimental models, the reduction of mitochondrial electron transport complexes activity in the kidney [90] produces an increase in FA release to the bloodstream and their posterior accumulation in other organs, particularly the liver [96]. Furthermore, altered lipid metabolism in the liver is observed during CKD. Liver FA synthesis increases, followed by FAS and ACC abundance elevation. The β-oxidation is also decreased via PGC-1α/PPARα/CPT1A reduction [99]. In the liver, CD36 is also increased [96], promoting FA and VLDL synthesis. Together, these data suggest that impairing mitochondrial β-oxidation, electron transport system activities, and biogenesis favor metabolic reprogramming. This enhances renal lipids uptake and its accumulation, promoting dyslipidemia in CKD.

## 5. Drugs and Their Drawbacks in CKD-Induced Dyslipidemia and Associated Cardiovascular Damage 

CKD is a syndrome that involves a variety of symptoms that must be treated to prevent their progression and the development of other complications. Managing CKD requires reducing cardiovascular risk, arterial hypertension, nephrotoxins, acidosis, and dyslipidemia [100]. Although different therapies are used during CKD treatment, some cautions must be considered. For example, RAAS inhibitors are utilized to slow the progression of CKD; however, recent studies have found that these inhibitors might cause hyperkalemia. In contrast, discontinuation of RAAS inhibitors is associated with an increased risk of initiation of dialysis and cardiovascular mortality [101]. 

Diuretics are the first-line treatment in acute decompensated HF; however, close monitoring is needed to avoid dangerous side effects in patients [8]. Potassium-sparing diuretics, such as amiloride, are used primarily in combination with thiazide or loop diuretics to prevent hypokalemia, and their diuretic effect is low. In contrast, aldosterone receptor antagonists and potassium-sparing diuretics can induce hyperkalemia, mainly in patients with renal dysfunction [8]. Furthermore, when renal function declines to eGFR <30 mL/min, thiazide diuretics are ineffective and cause hypokalemia and nocturia [101]. Although loop diuretics are the most common for HF and acute renal dysfunction, their short half-life and hemodynamic changes are their principal limitations. In addition, these diuretics might produce ototoxicity. Moreover, high doses of diuretics are often associated with increased serum creatinine and mortality, but data are inconclusive [102]. Vasodilators are often used in patients with preserved or elevated blood pressure to alleviate symptoms and improve hemodynamics; however, vasodilators increase stroke volume and cardiac output [103]. 

Other approaches to reduce dyslipidemia include pharmacological therapy with statins to lower cholesterol [104], ezetimibe, fibrates to reduce FA and triglycerides, niacin (HDL-increasing drug), and bile acid-binding resins [27,105,106]. Statins and fibrates are the most common treatments for dyslipidemia; however, these drugs could produce myopathy in the long term or in combination. Moreover, these drugs do not correct the lipid problem [107]. In this sense, managing dyslipidemia implies lifestyle modification and dietary interventions, such as reducing sugars, saturated fats, and salts [32]. Following the latter, treating severe hypercholesterolemia and very high-risk atherosclerotic CVD involves combining dietary and pharmacological therapies. However, its exclusive use is sometimes the most effective [108]. Therefore, searching for treatments that help significantly reduce dyslipidemia without modifying other parameters in patients is urgently needed. Moreover, it is necessary to use better alternatives that correct CKD symptoms without damaging other organs, preventing its progression, and avoiding the consumption of different drugs by the patients.

## 6. Curcuminoids

Turmeric (*Curcuma longa*) root has multiple properties, such as antioxidant and anti-inflammatory, showing beneficial effects on health [109]. Their principal active molecules are curcumin, bis-dimethoxy curcumin, demethoxycurcumin, and tetrahydro curcumin [110]. In addition, other synthetic curcumin derivates have shown high bioavailability and reabsorption [111].

### 6.1. Curcuminoids Bioavailability

Curcumin can be administered as concentrates or purified turmeric, curcuminoids (95%), or curcumin alone [112]. Most orally administered curcuminoids are excreted in the feces and urine. Therefore, very low is detected in blood plasma [113]. Low bioavailability has been linked to their lipophilic properties, difficulty absorbing water and acidic or neutral pH, and rapid metabolism to inactive metabolites [114]. The bioavailability of curcuminoids is a problem that prevents taking advantage of their benefits [115]. Therefore, different strategies have been implemented to increase curcuminoid availability [114]. 

Several formulations have been intended to enhance solubility and distribution to augment curcumin’s bioavailability [112]. Carriers or delivery systems’ synthetic compounds may increase curcuminoids’ bioavailability [114]. Some of the most common have included micelles, liposomes, phospholipids, microemulsions, nano-emulsions, emulsions, solid lipid nanoparticles, gelatin or polysaccharides, nanostructured lipid carriers, biopolymer nanoparticles and microgels [112,116,117]. Conjugated curcumin to phospholipidic carriers has increased its antioxidant capacities compared to when it is free [118]. Other strategies, such as liposomal curcumin (e.g., chitosan-coated curcumin and *Lallemantia iberica* seed gum nanoparticles), allow the correct encapsulation of curcumin and show an improvement in the mucoadhesive property [119]. The mucoadhesive property suggests prolonged adsorption in the gastrointestinal tract and has been shown to treat cancer effectively [120].

Recent techniques have been applied with outstanding results in different diseases. Magnetic nanoparticles provide multifunctional properties due to their controlled application. In this sense, magnetic-guide targeting in the delivery of curcumin diethyl γ-aminobutyrate, a carbamate prodrug of curcumin, has proved to be effective in cancer treatment due to its poor water solubility and improved delivery [121]. Other techniques include emulsion-based delivery systems used in the food industry to protect active ingredients against extreme conditions. One example is nanoemulsions formed with oil and emulsifiers that proved to augment the anti-inflammatory properties of curcumin in a model of 12-O-tetradecanoylphorbol-13-acetate-induced edema of mouse ear [122]. Curcuminoids’ complex formation is difficult due to their physicochemical features [114]. Therefore, more recent carriers have tried nanostructured lipid carriers with liquid and solid lipids. The ultrasonication method allows the encapsulation of whole turmeric into nanostructured lipid carriers. The technique can maintain turmeric’s physicochemical properties and stability.

Moreover, nanostructured lipid carriers protected gastric conditions, suitability, and safety for oral delivery, improved release control, and bioaccessibility compared with free turmeric [114]. The beneficial role of delivery systems in curcuminoids has been extensively proven. Alkaline conditions and organic solvents do not mimic those of the gastrointestinal tract and are very susceptible to auto-degradation. Therefore, careful experiments must be carried out [112,122,123]. Furthermore, more experimental and clinical studies are obligatory to prove curcumin’s beneficial effects in other models. Taken together, the studies showed that the availability of curcuminoids could be more feasible, and their clinical and basic research study is plausible and reproducible.

To date, the study of curcuminoid carriers to improve their bioavailability in CKD models has yet to be carried out. In current experimental models, the vehicles used include water [124], carboxymethyl cellulose [96], and yoghurt [125]. In patients, commercial curcumin is first given in juices, water [126], and capsules [127]. In line with this, curcumin is generally administered along with dietary lipids or lecithin to enhance its absorption. They are mainly found in food ingredients such as eggs, dairy products, or vegetable oils, facilitating tissue bioavailability and concentration [128] and making their use possible in clinical practice. It has been suggested in preclinical studies that curcumin could be a potent adjuvant to treat various disorders, including renal and cardiovascular damage and dyslipidemia [11].

### 6.2. Curcuminoids on CKD

Several factors might contribute to the progression of CKD, including parenchymal cell loss, chronic inflammation, fibrosis, and reduced regenerative capacity of the kidney [22]. The increased plasma creatinine and blood urea nitrogen (BUN) indicates that the kidney’s filtering capacity is diminished, and nitrogenous compounds are accumulating in the bloodstream [129]. In this context, curcumin could be a potential therapy to treat kidney damage at different levels. For example, at two different doses (60 and 120 mg/kg), curcumin improves renal function in rats with 5/6 nephrectomy (5/6NX), being the high doses the most effective [130]. Furthermore, curcumin reduces proteinuria, creatinine, and BUN levels by improving renal hemodynamics [130,131,132]. Similar results have been shown with tetrahydro curcumin at 1% given in food [133] (Table 1).

**Table 1:** Curcuminoids’ effects on chronic kidney disease (CKD) models.

Reference	Model	Compound	Dosage	Effect
[132]	5/6 nephrectomized Wistar rats	Curcumin	120 mg/kg	Reduces proteinuria, creatinine, and BUN serum levels, Improves renal function and blood pressure. Decreases oxidative stress through the Nrf2 pathway and monocyte infiltration by the reduction of MCP-1
[134]	Sprague–Dawley rats, renal injury induced by 0.25% adenine	Curcumin	37.5, 75 and 150 mg/kg	Decreases renal damage markers, inflammation (IL-1ß and IL-6), and fibrosis (caspase 3); and increases antioxidant indices (glutathione and super oxide dismutase).
[131]	5/6 nephrectomized Wistar rats	Curcumin	60 mg/kg	Reduces proteinuria, creatinine, BUN serum levels, and systolic pressure. Improve renal hemodynamics and mitochondrial respiration. Decrease oxidative stress, interstitial inflammation, and fibrosis.
[130]	5/6 nephrectomized Wistar rats	Curcumin	60 and 120 mg/kg	Reverts glomerular and systemic hypertension. Restores kidney tubular atrophy, reduces the mesangial area and mesangial cells proliferation, prevents the expansion of the glomerular matrix
[135]	Dahl salt-sensitive rats, nephrosclerosis induced by salt	Curcumin	10 mg/kg	The antifibrotic effect could be through the inhibition of histone acetylation (H3K9)
[133]	5/6 nephrectomized Sprague–Dawley rats	Tetrahydro curcumin	1% in food	Improves the expression of antioxidant enzymes in the kidney, decreases renal apoptosis and fibrosis and ameliorates proteinuria, hypertension, and cardiac hypertrophy.
[136]	Mice with unilateral ureteral obstruction	Bisdemethoxycurcumin	100 and 200 mg/kg	Reduces fibrosis throw fibroblast apoptosis

BUN: Blood urea nitrogen, Nrf2: Nuclear factor erythroid 2-related factor 2, H3K9: histone 3 lysine 9, IL: interleukin, MCP-1: monocyte chemoattractant protein-1.

During exercise or strenuous physical activities, water excretion and protein metabolism increase, which could further damage the kidney during CKD [137]. Curcumin (75 mg/kg/day) prevented increased creatinine, proteinuria, and BUN levels in a renal damage model induced by adenine and aerobic exercise stress [138]. Another critical aspect in managing CKD is balancing the diet because hypercaloric diets (western diets) produce metabolic stress, dyslipidemia, and severe damage to kidney tissue [139]. Curcumin (100 mg/kg) administration in mice with CKD exposed to a western diet showed a reduction in the urine ratio of albumin-creatinine compared to a control diet [140]. Thus, curcumin could be used as a potential treatment to prevent the consequences of diet management in CKD patients (Table 1).

The improvement in renal function by curcumin also prevents tissue degeneration. Curcumin (120 mg/kg) reversed renal tubular atrophy in 5/6NX rats by promoting the reduction of the mesangial area and mesangial cell proliferation, avoiding the expansion of the glomerular matrix [130]. Moreover, curcumin, combined with other natural compounds at different concentrations, reduced the expression of smooth muscle actin (α-SMA) in NFK-49F cells proving its antifibrotic effect [141] (Table 1). At a 60 mg/kg dose, curcumin prevented renal hypertrophy by reducing interstitial fibrosis and 50% of glomerular and global sclerosis [131,142]. The same effect was observed with tetrahydro curcumin at 1% in food, which reduced approximately 20% of renal fibrosis [133]. The antifibrotic effect of curcumin has been associated with the inactivation of the mammalian target of rapamycin/HIF-1α/vascular endothelial growth factor (mTOR/HIF-1α/VEGF) signaling pathway in vitro (10 and 20 µM doses of curcumin) [136] and in vivo (100 and 200 mg/kg doses of curcumin) [143]. Furthermore, in a nephrosclerosis salt-sensitive model, the antifibrotic effect of curcumin was attributed to the inhibition of histone acetylation in histone 3 lysine 9 (H3K9) [135] (Table 1).

On the other hand, it has been well-described that curcumin has an antioxidant effect [144]. For instance, the minimum curcumin antioxidant dose of 60 mg/kg is enough to induce the Keap1/Nrf2 signaling pathway and promote nuclei translocation of Nrf2. This increases the expression, protein levels, and activity of antioxidant enzymes like glutathione peroxidase, glutathione reductase, and superoxide dismutase 1 in the 5/6NX and adenine models [130,131,132,134]. In vitro studies showed that curcumin and demethoxycurcumin decrease ROS levels and MDA content, and increase superoxide dismutase activity, avoiding apoptosis in advanced glycation end products-induced oxidative stress in mesangial cells [145]. In line with this, it has been demonstrated in the 5/6NX model that curcumin reduces the NOX activity in the renal cortex and proximal tubules [132,133]. Other authors hypothesized that curcumin decreases oxidative stress by reducing endoplasmic reticulum stress, preventing apoptosis in podocytes, and improving renal function [146]. These mechanisms were associated with regulating the mitogen-activated protein kinase/extracellular signal-regulated kinase 1/2 (MAPK/ERK1/2) signaling pathway [147]. Thus, one of the principal mechanisms mediated by curcumin is its ability to reverse oxidative stress by avoiding ROS overproduction (Table 1). 

Curcuminoids also have anti-inflammatory effects in CKD. For instance, in the 5/6NX model, curcumin at 60 mg/kg reduced the interstitial inflammation in the remnant kidney, falling from 50 to 20 macrophages per field and preventing monocyte chemoattractant protein-1 (MCP-1) overexpression [131,132]. In addition, curcumin reduced plasmatic concentrations of TNF-α and IL-6 [140]. Reducing all mentioned cytokines decreases kidney inflammation and stabilizes kidney function. In the cisplatin model, an acute model, intraperitoneal curcumin at 100 mg/kg, prevented macrophage infiltration in the kidney at 24 h. The beneficial effect was achieved by blocking macrophage inducible Ca^2+^-dependent lectin receptor (Mincle), diminishing spleen tyrosine kinase (Syk)/NF-κB signaling and, therefore, reducing IL-1β, TNF-α, IL-6, and MCP-1 expression [148]. Concerning NF-κB signaling, its canonical activation is given by p65/p50 heterodimer [149], which translocates to the nucleus to induce the expression of proinflammatory cytokines like TNF-α, IL-1, IL-2, IL-6; adhesion molecules such as intercellular adhesion molecule (ICAM)-1, vascular cell adhesion molecule (VCAM)-1, E-selectin, chemokines (e.g., IL-8, MCP-1, regulated on activation, normal T cells expressed and secreted (RANTES)), and inducible enzymes such as cyclooxygenase (COX) 2 and inducible nitric oxide synthase (iNOS) [150]. On the other hand, curcumin also avoids inflammation through arachidonic acid hydrolyzation, inhibiting phospholipase A2 (cPLA2) phosphorylation and decreasing COX1 and COX2 [142]. In the immune nephritis model, 1 g/kg of curcumin for 15 days reduces the periglomerular and perivascular lymphocyte infiltration [151] (Table 1).

In CKD patients, curcuminoid’s effects are poorly investigated; however, it has been found that in mononuclear cells isolated from CKD patients, 1–3 mM of curcumin decreases the secretion of Il-6 and IL-1ß and its procoagulant activity [152] (Table 2). A similar effect was seen in the plasma of CKD patients treated with 1 g per day of Meriva^®^ (demethoxycurcumin), which reduced lipid peroxidation and plasma pro-inflammatory mediators like MCP-1, IFN-γ, and IL-4 [153]. In hemodialyzed patients, 2.5 g of turmeric (the whole root) decreased NF-κB in mononuclear cells, TNF-α plasma levels, and regulated gut microbiota [126,154,155]. In the early stages of renal failure, curcuminoids in combination with *Boswellia serrata* influenced IL-6 and prostaglandin E2 plasma concentrations, avoiding CKD progression [156]; however, more studies are required to determine the mechanisms involved in improving renal function by curcuminoids (Table 2).

**Table 2:** Curcuminoids’ effects on CKD patients.

Reference	Model	Compound	Dosage	Effect
[152]	CKD patients’ mononuclear cell culture	Curcumin	1 and 3 mM	Decreases secretion of IL-6 and IL-1β. Decreases the procoagulant activity of mononuclear cells.
[127]	Clinical trial, CKD patients with coronary angiography or angioplasty	Curcumin	1.5 g	There are no significant changes
[153]	Clinical trial, stage 3 to 4 CKD patients	Meriva^®^	1 g/day	Increases plasma creatinine, decreases eGFR, and changes microbiota diversity. Reduces plasma pro-inflammatory mediators (MCP-1, IFN-γ, and IL-4) and lipid peroxidation.
[156]	Clinical trial, CKD patients with coronary angiography	Curcuminoids	1.5 g/day	Preserve changes in eGFR preventing post-contrast acute kidney injury.
[157]	Clinical trial, non-dialysis CKD patients	Curcuminoids and *Boswellia serrata*	824 and 510 mg/day, respectively	There was a time effect and time x compliance interaction effect for IL-6
[158]	Clinical trial, stage 2 to 3 CKD patients	Curcuminoids and *Boswellia serrata*	824 and 510 mg/day, respectively	There was a group effect and a trend for group × time interaction for prostaglandin E2.
[154]	Clinical trial, hemodialyzed CKD patients	Turmeric	2.5 g	Decreases in NF-κB mRNA expression in mononuclear cells and in plasma high-sensitivity CRP levels
[126]	Clinical trial, hemodialyzed CKD patients	Turmeric	2.5 g	Decreases in pCS plasma levels, suggesting gut microbiota regulation
[155]	Clinical trial, hemodialyzed CKD patients	Turmeric	2.5 g	Reduces TNF-α plasma levels

MCP-1: monocyte chemoattractant protein-1, IFN-γ: interferon-gamma, NF-κB: nuclear factor-kappa B, CRP: C-reactive protein, pCS: p-cresyl sulfate, TNF-α: tumor necrosis factor-α, IL: interleukin, eGFR: estimated glomerular filtration.

### 6.3. Effects of Curcuminoids on CKD and Associated Cardiovascular Damage

The protective role of curcuminoids has been probed in preclinical models of CKD and concurrent cardiovascular alterations [159]. For example, in the heart of nephrectomized rats, curcumin prevented macrophage infiltration and reduced the inflammasome component levels NLRP3, apoptosis-associated speck-like (ASC), and caspase-1, preventing inflammasome activation. The latter avoided IL-1β release, reducing inflammatory levels [160]. Administration of curcumin at doses of 60 or 120 mg/kg/day in rats after 5/6NX with or as a prophylactic treatment reverted glomerular and systemic hypertension and improved renal function and structure. The beneficial effects were similar to those of enalapril, an inhibitor of the angiotensin-converting enzyme 2 [130]. In addition, the chronic administration of Theracurmin^®^ (100 mg/kg/day gavage for 5 weeks) in the 5/6NX rat model improved ventricular function and avoided fatal consequences such as heart hypertrophy and interstitial fibrosis by reducing beta myosin heavy chain (ß-MHC) and Col I levels [160]. In the same model, the administration of tetra hydro curcumin, at a dose of 1% in the food per 9 weeks, showed a decrease in systolic and diastolic blood pressure associated with hypertrophy prevention [133]. In line with this, ventricle hypertrophy and dilatation were prevented through the reduction of glycogen synthase kinase 3 beta (pGSK-3ß), ß-catenin and nuclear factor of activated T-cells (NFAT) levels [142] (Table 3).

**Table 3:** Curcuminoids’ effects on chronic kidney disease (CKD)-induced cardiovascular damage.

Reference	Model	Compound	Dosage	Effect
[138]	Sprague–Dawley rats, renal injury induced by 0.25% adenine	Curcumin	75 mg/kg	Prevents increased creatinine, proteinuria, and BUN levels in CKD during exercise. Prevents the increase in systolic blood pressure and increases the activity of antioxidant enzymes. Decreases fibrosis and inflammation.
[140]	C57Bl/6 mice with 5/6 nephrectomy and western diet	Curcumin	100 mg/kg	Reduces the urine albumin-creatinine ratio and decreases arteriosclerotic lesions. Improve glucose tolerance, and decrease inflammation and blood pressure.
[142]	5/6 nephrectomized Sprague–Dawley rats	Curcumin	150 mg/kg	Attenuates cardiac hypertrophy and remodeling through the reduction in pGSK-3ß, ß-catenin, and NFAT levels.
[160]	5/6 nephrectomized Sprague–Dawley rats	Theracurmin^®^	100 mg/kg/day	Improves ventricular function and avoids heart hypertrophy and interstitial fibrosis by reducing ß-MHC and collagen type 1
[161]	5/6 nephrectomized Wistar rats	Curcumin	120 mg/kg	Decreases principal CKD biochemical markers. Prevents ventricular hypertrophy and decreases ischemic events and oxidative stress in heart tissue.
[162]	5/6 nephrectomized Wistar rats	Curcumin	120 mg/kg	Prevents the tissular remodeling process reducing MMP-2, the activity of gelatinase, and the activation of the IP3K/AKT/ERK signaling pathway

BUN: Blood urea nitrogen pGSK-3ß: glycogen synthase kinase 3 beta, NFAT: nuclear factor of activated T-cells, ß-MHC: beta myosin heavy chain, MMP-2: matrix metalloproteinase 2, PI3K/AKT/ERK: phosphatidylinositol 3 kinase/protein kinase B/extracellular signal-regulated kinase.

In the 5/6NX model, 100 mg/kg/day of curcumin for 16 weeks decreased arteriosclerotic lesions [140], while 120 mg/kg reduced necrotic lesions in mice exposed to a western diet [161] by preventing the tissular remodeling process through the reduction of matrix metalloproteinase 2 (MMP-2) and the activity of gelatinase. These processes might be related to activating the phosphatidylinositol 3 kinase/protein kinase B/extracellular signal-regulated kinase (IP3K/AKT/ERK) signaling pathway [142,162]. 

Therefore, the studies mentioned above suggest curcumin could also be used as an alternative adjuvant or therapy to prevent cardiovascular side effects related to hypertrophy, cardiac remodeling, and ventricular function during CKD (Table 3).

### 6.4. Regulation of Dyslipidemia by Curcuminoids in CKD 

The effect of curcumin on dyslipidemia has been determined in diabetes and obesity models, demonstrating beneficial results [163]. Since the liver is the main lipid metabolism organ, most studies have used it to assess the curcumin effect on dyslipidemia in this organ. For instance, curcumin prevents hepatic lipotoxicity in diabetic and obese models, modulating the metabolism of cholesterol by forming bile acids and increasing the oxidation of fatty acids. At the same time, curcumin increases serum HDL and the activity of lipases that prevents the increased uptake of fatty acids [163]. Recent studies in obese rats treated with curcumin (80 mg/kg) and *Garcinia mangostana* (400 mg/kg) for 6 weeks showed that curcumin reduces oxidative stress, increases HDLc, and decreases LDLc sera levels [164]. In the high-fat diet induced-diabetic mice model, treating tetra hydro curcumin at 100 mg/kg for 12 weeks decreased the renal damage markers and cholesterol and triglycerides levels. The authors proposed that tetra hydro curcumin deactivates the renin-angiotensin system, which reduces oxidative stress. The reduction in oxidative stress causes a decrease in lipid levels, attenuating dyslipidemia [165]. In diabetic patients, a meta-analysis suggests that curcumin supplementation could lower LDL, TG, and TC levels in complicated type two diabetes [166]. Furthermore, in metabolic syndrome patients, a syndrome associated with diabetes development, 200 mg/day of curcumin reduces HDLc, LDL, TG, and TC serum levels [167].

In the obesity and diabetes protocols, it also has been reported that curcumin decreased dyslipidemia, attributed to its binding to lipids in the intestine [168]. The proposed molecular mechanism is mediated by cyclic adenosine monophosphate (cAMP) responsive element binding protein (CREB)/PPAR signaling pathway, which increases cAMP concentrations to promote lipid oxidation [169]. In adipose tissue, curcumin inactivates the AKT/mTOR signaling pathway, preventing adipogenesis, FA uptake, and triglyceride formation [170]. Curcumin also increased paraoxonase 1 activity and lipoprotein lipase abundance in plasma and the liver, promoting lipoprotein lipids hydrolysis and their oxidation in the tissues [124,125]. In an in silico study, curcumin showed a particular interaction with ADIPOQ and PPARG genes, both are closely related to lipid metabolism [171]. In C57BL/6J mice with renal injury induced by a high-fat diet, the treatment with bisdemethoxycurcumin at 20 and 40 mg/kg avoided lipid accumulation, oxidative stress, and improved plasma lipid levels through Nrf2/Keap1 [172]. Thus, curcumin has an antihyperlipidemic role in CKD related to these pathways.

Few studies have evaluated the effects of curcumin on serum lipids during CKD. Currently, some attempts have found that curcumin modulates lipid metabolism in renal tissue and decreases serum and liver triglycerides, cholesterol, free FA, and LDL levels. In experimental models such as the 5/6NX, the administration of 75 mg/kg of curcumin for 11 weeks corrected the serum lipid profile by decreasing LDL, total cholesterol, and total triglycerides and increasing HDL levels [173], suggesting that curcumin has a positive effect on serum lipids unbalance. Supporting the latter, in the adenine CKD model, curcumin treatment with 100 mg/kg increased HDL cholesterol while decreasing total cholesterol, triglycerides, LDL cholesterol, VLDL, and non-esterified FA (NEFA) [174]. The authors also found that triglycerides and NEFA levels in the liver decreased, but cholesterol levels increased. This could be partly explained because increased serum HDL concentrations led to increased cholesterol uptake in the liver, which produced further metabolization and elimination via the bile [174].

**Figure 3:**
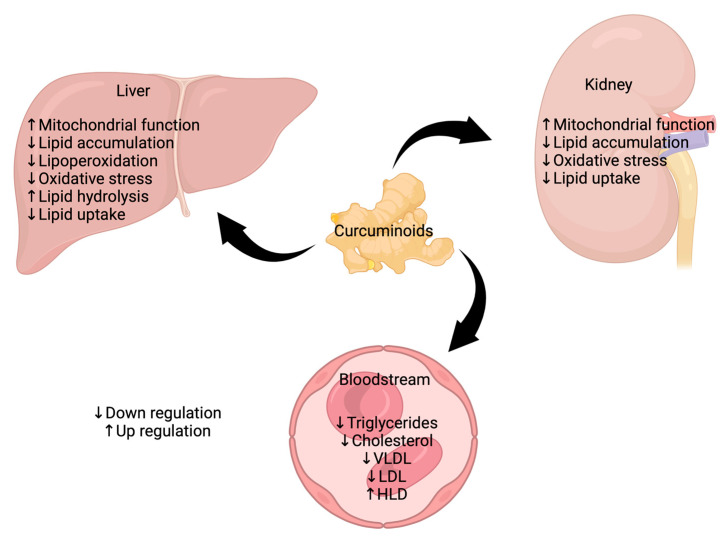
Regulation of lipids by curcuminoids in chronic kidney disease (CKD). Different beneficial effects can be attributed to curcuminoids in the improvement of CKD. For example, curcuminoids may restore the imbalance of lipids in the bloodstream, such as triglycerides, cholesterol, VLDL, LDL, and HDL levels. In the liver, curcuminoids can improve mitochondrial function and reduce lipid accumulation, lipoperoxidation, oxidative stress, and lipid uptake, as well as increase lipid hydrolysis. Finally, in the kidney, curcuminoids have also improved mitochondrial function and reduced lipid accumulation, oxidative stress, and lipid uptake. The restoration of these mechanisms together produces the improvement of CKD or the avoidance of its progression toward other detrimental effects in the organism. VLDL = very low-density lipoproteins, LDL = low-density lipoproteins, HDL = high-density lipoproteins.

Interestingly, the authors reported that the atherogenic and the coronary risk index also decreased, suggesting that the correction of lipid profile by curcumin influences cardiovascular alterations [174]. According to the latter, the decrease of LDL and VLDL reduces the formation of atheroma, a severe consequence of dyslipidemia [175]. Recently, our group determined a possible mechanism in 5/6 NX-induced CKD. We found that curcumin corrects dyslipidemia by improving renal mitochondrial β-oxidation function. This prevents lipid accumulation, its distribution, and FA uptake by the liver [96], suggesting that the kidney is the origin of dyslipidemia (Table 4 and Figure 3). 

**Table 4:** Curcuminoids’ effects on CKD dyslipidemia.

Reference	Model	Compound	Dosage	Effect
[173]	5/6 nephrectomized Sprague–Dawley rats	Curcumin	75 mg/kg	Decreases LDL, total cholesterol, and total triglycerides
[174]	Sprague–Dawley rats, renal injury induced by 0.25% adenine	Curcumin	100 mg/kg	HDL cholesterol increases and decreases total cholesterol, triglycerides, LDL cholesterol, VLDL, NEFA, atherogenic index, and the coronary risk index. In the liver, it increases cholesterol and decreases triglycerides and NEFA.
[176]	Meta-analysis	_	_	Reduces total cholesterol and TNF-α. Not confirm significant changes in triglyceride, LDL-cholesterol, HDL-cholesterol, and CRP.
[177]	Clinical trial, patients with nondiabetic proteinuria CKD	Curcumin	320 mg/day	Attenuates lipid peroxidation and enhances the antioxidant capacity.
[172]	C57BL/6J mice, renal injury induced by a high-fat diet	Bisdemethoxycurcumin	20 and 40 mg/kg	Decrease renal injury markers, inflammatory cytokines, and tissue fibrosis. Decreases body and white adipose weight, serum glucose, insulin, TC, TG, and HDL-C levels. Increases antioxidant activity and decreases lipid accumulation through Keap1/Nrf2.

LDL: low-density lipoproteins, HDL: high-density lipoprotein, VLDL: very low-density lipoprotein, NEFA: non-esterified fatty acids, Keap-1: Kelch-like-ECH associated protein-1, Nrf2: nuclear factor erythroid 2-related factor 2, TC: total cholesterol, TG: triglycerides, CRP: C-reactive protein, TNF-α: tumor necrosis factor-α.

## 7. Conclusions

CKD is characterized by a progressive decline in renal function, which triggers several pathological mechanisms, resulting in CVD consequences. Among them, dyslipidemia plays a crucial role in CVD development. Dyslipidemia is strongly related to oxidative stress, inflammation, metabolic reprogramming, and fibrosis in renal tissues. These pathological processes worsen renal disease and increase the plasmatic lipid levels, which results in metabolic lipid alterations in other tissues, like the liver and heart. The current drugs used to overcome these pathophysiological mechanisms produce side effects and are only sometimes optimal for all CKD types and populations. 

Recent studies have shown that curcuminoids may improve lipid disorders in diabetes and obesity. Moreover, a potential therapy for CKD-induced hyperlipidemia has been given. The administration of curcuminoids reverses CKD-induced metabolic reprogramming, avoiding the decrease in β-oxidation and preventing mitochondrial damage. Therefore, curcuminoids might avoid the accumulation of lipids in renal tissue. In addition, curcuminoids reverse increased FA uptake and synthesis, closely related to the decrease in oxidative stress and pro-inflammatory and pro-fibrotic processes in the kidney, reducing the release of lipids into the bloodstream. The curcuminoid’s protection also might decrease the pathological processes associated with the development of CVD during CKD by regulating dyslipidemia. Although the decrease in cardiovascular damage has been shown in several CKD experimental models, further investigation should be generated to determine the effects of curcumin in patients with CKD.

## Data Availability

Not applicable.
